# Comparative removal of hazardous cationic dyes by MOF-5 and modified graphene oxide

**DOI:** 10.1038/s41598-022-19550-5

**Published:** 2022-09-12

**Authors:** Ali Akbar Mohammadi, Soheila Moghanlo, Malihe Samadi Kazemi, Shahram Nazari, Seid Kamal Ghadiri, Hossein Najafi Saleh, Mika Sillanpää

**Affiliations:** 1 Department of Environmental Health Engineering, Neyshabur University of Medical Sciences, Neyshabur, Iran; 2 Department of Environmental Health Engineering, Zanjan University of Medical Sciences, Zanjan, Iran; 3 Department of Chemistry, Faculty of Sciences, Bojnourd Branch, Islamic Azad University, Bojnourd, Iran; 4 Department of Environmental Health Engineering, Khalkhal University of Medical Sciences, Khalkhal, Iran; 5 Department of Environmental Health Engineering, School of Public Health, Shahroud University of Medical Sciences, Shahroud, Iran; 6 Environmental and Occupational Health Research Center, Shahroud University of Medical Sciences, Shahroud, Iran; 7 Department of Chemical Engineering, School of Mining, Metallurgy and Chemical Engineering, University of Johannesburg, P. O. Box 17011, Doornfontein, 2028 South Africa; 8 International Research Centre of Nanotechnology for Himalayan Sustainability (IRCNHS), Shoolini University, Solan, Himachal Pradesh 173212 India

**Keywords:** Environmental sciences, Chemistry

## Abstract

Among cationic dyes, malachite green (MG) is commonly used for dying purposes and also as an inhibitor in aquaculture, food, health, and chemical industries due to its cytotoxic effects. Therefore, MG removal is essential to keep the ecosystem and human health safety. Adsorption is a viable and versatile option and exploring efficient adsorbents have high priority. Herein, MOF-5 and aminated corn Stover reduced graphene oxide (ACS-RGO) of typical adsorbents of metal–organic-frameworks (MOFs) and carbon-based classes were studied for MG removal. MOF-5 and ACS-RGO had a specific surface area and total pore volume of 507.4 and 389.0 m^2^/g, and 0.271 cm^3^/g and 0.273 cm^3^/g, respectively. ACS-RGO was superior for MG adsorption and the kinetic rate coefficient for ACS-RGO was ~ 7.2 times compared to MOF-5. For ACS-RGO, MG removal remained high (> 94%) in a wide range of pH. However, dye removal was pH-dependent for MOF-5 and increased from ~ 32% to ~ 67% by increasing pH from 4 to 12. Increasing dye concentration from 25 mg/L to 100 mg/L decreased adsorption by MOF-5 and ACS-RGO for ~ 30% and 7%, respectively. Dye removal was evident in a few tens of seconds after adding ACS-RGO at doses above 0.5 g/L. A significant loss of 46% in adsorption was observed by decreasing MOF-5 mass from 1 to 0.1 g/L. ACS-RGO removed MG in multilayer with an exceptional adsorption capacity of 1088.27 mg/g. In conclusion, ACS-RGO, and MOF-5 showed promising kinetic rates and adsorption capacities toward MG.

## Introduction

Dyes are substantially consumed by textile and other industries and hundreds of thousands of different dye species are commercially available. In the long list of industries, the textile industry accounts for the principal consumption of dyestuffs^1^. Dye utilization percentage due to the low fixation rate is usually low and hence about 50% of dye substances are usually wasted as wastewater^2^. The low biodegradability and heterocyclic structure of many dyes prevent efficient biodegradation of these substances in industrial wastewater treatment systems. As implied from the name, dyes are highly visible and their presence in aquatic environments is not only aesthetically unpleasant but also affects the ecosystem due to their toxicity on aquatic life and by preventing natural photosynthesis.

The cytotoxicity feature and anti-parasitic properties of MG cause the extensive utilization of MG in aquaculture, food, health, and chemical industries^3^. However, some studies indicated the detrimental effects of ingesting MG on the DNA of model animals^4^.

Cationic dyes in environment are toxic, carcinogenic, and mutagenic can cause health problems such as dysfunction of the liver, brain and central nervous system^5^. Accordingly, removal of dyes in wastewater before discharging to environment is necessary. Various technic such as photocatalytic degradation^6^, membrane filtration^7^, ion-exchange^8^, electrolysis^9^, biological processes^10^ and adsorption^11^ are used to treatment wastewater and to reduction dye concentration before discharge to environment.

Among these methods adsorption is a favored technique due to the numerous advantages it has over different methods available for pollution control. Easy design and operation, environmental benign nature, high efficiency under low pollutant concentration, availability of adsorbents, the flexibility of the process make adsorption an interesting and versatile option. Numerous studies oriented in the past years to develop new structures to maximize the advantages of adsorbent materials^12^. In recent years, many materials have been used as adsorbents in industrial dyed wastewater treatment, for example agricultural waste^12^, natural mineral^13^, activated carbon^14^, zeolite^15^, Graphene oxide^16^ and Metal–organic-frameworks^17^. Metal–organic-frameworks (MOFs) are an exceptional class of porous materials composed of inorganic (metal ions or clusters) and organic (linker or ligand) components. MOFs are highly crystalline with a huge internal surface area and pore volume. Furthermore, they are highly tunable to design and functionalize, and hence they are recognized as materials of interest in applied sciences^18^.

MOF-5 (chemical formula: Zn_4_O_13_-(C_8_H_4_)_3_), also known as IRMOF-1, is a well-known MOF due to its unique porosity and thermal stability^19^. MOF-5has three-dimensional structure consisting of terephthalic acid and Zn_4_Oclusters^20^. The stability and flexible functionality of MOF-5 have attracted attention in drug delivery^18^, molecular storage and separation^21^, sensing^20^, and water remediation contaminated by antibiotics^22^, nitrate^23^, methyl orange^24^ andradionuclide^25^.

Graphene is the thinnest material produced so far and the simplest form of carbon that exists in two-dimensional form. Due to the exceptional mechanical, electrical, thermal, biological, optical, and other physicochemical properties of graphene and its derivatives, the graphene family experienced massive and rapid growth in the fields of electronics, biosciences, and environment^26^.

Graphene oxide (GO) is a scalable derivate of graphene that is highly hydrophilic and rich indifferent functional groups such as carboxyl, hydroxyl, and epoxy. Reduced graphene oxide (RGO)is also a type of graphene that the presence of abundant hydroxyl functional groups has caused the hydrophobicity significantly^27,28^. The presence of functional groups and the adaptability of being composite, functionalized, and decorate, surged interest toward graphene family for treatment purposes such as adsorption, catalysis, membrane separation, ion exchange, dialysis, etc.^29,30^. As an adsorbent, graphene family has been studied and promising results obtained for the removal of heavy metals^31^, pesticides^32^, antibiotics^33,34^, and other emerging contaminants. The aforementioned functional groups on graphene surface play as active sites for modification by basic moieties like amines. In some cases, GO needs more modification to improve the adsorptive properties for target contaminants. RGO, on the other hand, has a simpler and more predictable behavior for surface modification because it contains only one type of functional group. Of the variety of techniques proposed for amine functionalization such as plasma electron beam, hydrothermal reaction, and Leuckart reaction, the hydrothermal approach is most common. Fanget al prepared GO-NH_2_Nano sheets with a surface area of 320 m^2^/g and rapid adsorptive properties for cobalt cations^35^. Awad et al. improved GO adsorption properties for mercury (II) through the incorporation of different functional groups using solvothermal methods. They reported a premium removal of 100% when GO was functionalized by carboxylic acid^36^. Viana et al. functionalized graphene oxide with diethylenetriamine using a microwave-assisted method and then prepare a hydrogel adsorbent. The prepared hybrid hydrogel was finally used as an efficient adsorbent for methylene blue^37^. In the previous study, we reported a green approach for valorizing corn Stover biomass to reduced graphene oxide (RGO). A post-treatment approach was then applied to convert RGO to aminated graphitic carbonaceous structure (ACS-RGO). Afterward, ACS-RGO was used as a promising adsorbent for antibiotic tetracycline where a high 132.9 mg/g adsorption capacity was obtained at pH 7.4 ^38^.

In this regard for the first time, the goal was to evaluate the dye adsorption properties of MOF-5 as a representative of hybrid structures and ACS-RGO as a typical member of carbonaceous materials. Therefore, this study was initiated by a comparative analysis for adsorption efficiency and rate of dye removal by kinetic modeling. The study then oriented toward the detailed analysis of the effects of operating variables i.e. pH, adsorbent dose, MG concentration, and mixing time. To elucidate the mechanism of adsorption, and to compare the adsorption capacity of herein materials, the equilibrium of the adsorption system was investigated.

## Materials and methods

### Chemical and reagents

Chemicals used in the study were of analytical grade and purchased from Merck and Sigma Aldrich companies. Adsorbate, malachite green oxalate = Basic Green 4, chemical formula = C_52_H_54_N_4_O_12_, MW = 927, the wavelength of maximum absorbance = 624 nm was purchased from Sigma Aldrich. Terephthalic acid (TPA) = C_8_H_6_O_4,_ molar mass = 166.13 g/mol, N,N’-dimethylformamide (DMF), and zinc nitrate hex hydrate were used without further modification.

### Adsorbents preparation and characterization

MOF-5 was prepared by the protocol described in the literature^39^. In brief, 0.595 g zinc nitrate hexahydrate and 0.111 g TPA were dissolved in 20 mL DMF. The clear solution was then sonicated at 35 kHz for 10 min and transferred to a Teflon-lined autoclave where it was heated for 24 h at 135 °C. After cooling at room temperature, the white precipitates were collected by centrifuge and washed with fresh DMF, and dried overnight.

A detailed protocol for the synthesis of aminated corn Stover-based reduced graphene oxide (ACS-RGO)including collecting Agro waste materials, washing, calcination, activation, thermo-chemical treatment, and surface modification with amine was described in our previous work^38^.

The presence of functional groups on the surface of the adsorbent were ascertained by recording Fourier-transform infrared spectroscopy (FTIR) by a Thermos Nicolet, Avatar 370 spectrophotometer.

The surface morphology and crystal texture were studied by field emission scanning electron microscopy (FE-SEM) using MIRA3 TESCAN, Czech Republic. The structure of crystals was analyzed by X-ray diffraction (XRD) usingUnisantis S.A, XMD300 model, Geneva, Switzerland, with Cu-kα as source radiation at wavelength 0.154 nm), over the range of 10° to 80°. Pore volume, specific surface area (SSA), and pore sizes of adsorbents were examined by the nitrogen sorption using BELSORP-mini-II (BEL Japan, Inc.).

### Adsorption experiments

The present study was designed to compare the dye adsorptive characteristics of porous materials from two interesting classes, MOFs and carbonaceous structures. When MOF-5 and ACS-RGO were synthesized and characterized, the experimental study began with a comparative analysis of MG removal using adsorption capacity and kinetic modeling. The kinetic parameters for dye removal were estimated by non-linear regression models. Next, parametric evaluation and equilibrium modeling were conducted. Effect of pH (4–12), initial dye concentration (25–100 mg/L), mixing time (2–60 min), and adsorbent dose (0.1–1 g/L) were surveyed in parametric evaluation step.

All the experiments were performed in batch mode, at room temperature, and under mixing at 250 rpm. After adsorption, materials were separated by centrifuge and dye concentration in supernatants determined by UNICO UV-2100 spectrophotometric method at 626 nm. As presented in Fig. S1, it is noticeable that the light adsorption intensity for MG is pH-dependent and adsorption intensity was reduced over 50% by increasing pH from 4 to 12. Fig. S1 shows the dye intensity for a solution containing 50 mg/L MG at pH 4 and pH 12. Dye removal efficiency (µ%) was calculated for each run by the difference between dye concentration after (C, mg/L) and before adsorption (C_0_, mg/L):

1 $$\upmu \left(\%\right)=\frac{{\mathrm{C}}_{0}-\mathrm{C}}{{\mathrm{C}}_{0}}\times 100$$

The capacity of adsorbents in any time (q_t_, mg/g) was calculated according to the mass of adsorbents in the solution (m, g), the volume of solution (V,L), initial dye concentration (C_0_, mg/L) and dye concentration at any time (C_t_, mg/g)^40^:

2 $${\mathrm{q}}_{t}=\frac{{(\mathrm{C}}_{0}-{\mathrm{C}}_{\mathrm{t}})}{\mathrm{m}}\times \mathrm{V}$$

### Adsorption modeling

Adsorption modeling is a suitable approach for obtaining basic information required to scale up the system. Kinetic models describe the rate of adsorption system and are insightful in identifying the rate-limiting step in the process. Isotherm models, on the other hand, describe the equilibrium state of the sorption and provide a useful tool for comparing adsorbents for a specific contaminant and also the utilization rate of adsorbents in real treatment systems. Kinetic data was collected by performing the adsorption experiments at different mixing times. Three common non-linear models i.e. pseudo-first-order (PFO), pseudo-second-order (PSO), and intraparticle diffusion model (IDM) were fitted to the kinetic data.

The equilibrium data was collected by conducting adsorption experiments on solutions withthe different initial dye concentrations in the range of 50–300 mg/L. The adsorbent capacities were then calculated and modeled using Langmuir, Freundlich, and Javanovich models.

### Regeneration study of ACS-RGO

To conduct the regeneration tests, the well saturated ACS-RGO was contacted with 0.1 M hydrochloric acid solution (0.1 mol/L HCl) as eluting agent. The adsorbed MG dye desorbed under 2 h’ agitation at 250 rpm. After separating ACS-RGO, the MG concentration in the supernatant was measured. ACS-RGO then rinsed with distilled water twice and used for the next MG removal cycle under optimal conditions. MG desorption ratio was determined using the following equation:

3 $$Desorptionratio\left(\mathrm{\%}\right)=\frac{amount\,of\,MGdyedesorbed}{amount\,of\,MGdyeadsorbed}\times 100$$

## Results and discussion

### Adsorbent characteristics

**Figure 1:**
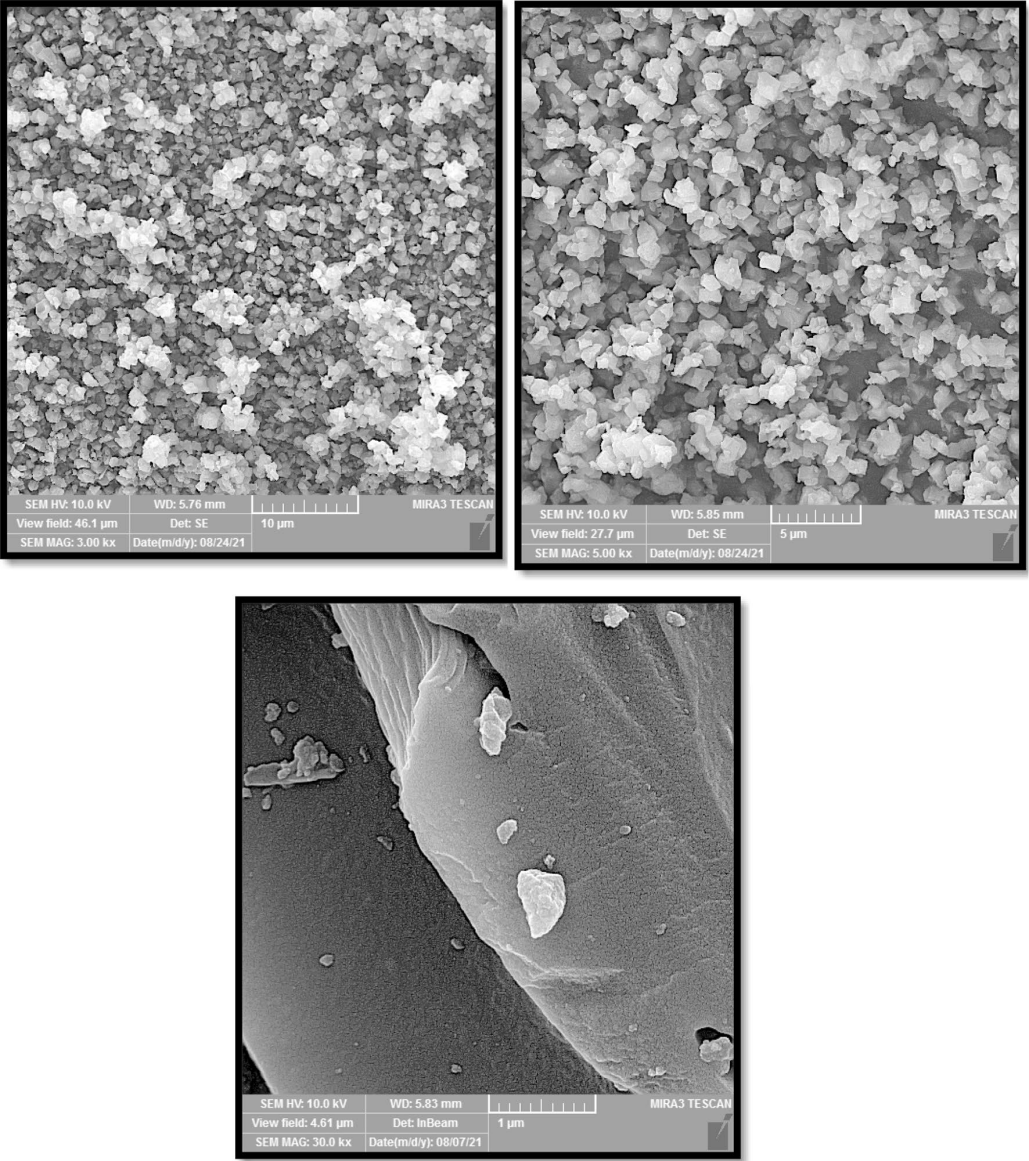
The FESEM images of (left) MOF-5 crystals with two magnification, and (right) ACS-RGO Nano sheets.

The FESEM images in Fig. 1 (left) show the morphology of MOF-5 crystals. As seen, the crystals have a trapezoidalcross-section with a length of up to ~ 50 µm. The width of MOF-5 crystals varied from a few micrometers up to ~ 20 µm. Studies recorded different morphologies for MOF-5 crystals. For instance, Mirsoleimani-Azizi et al. synthesized cube MOF-5 with an average diameter of 552 nm using zinc acetate and under room temperature^22^. Jin Son et al. synthesized cube MOF-5 of 5–25 μm in crystal size using a rapid (~ 30 min) sonochemical method^41^. SikChoi et al. synthesized uniform cubic MOF-5 crystals of 20–25 μmin size through a microwave heating solvothermal route using 1-methyl-2-pyrrolidone as a solvent^42^. Zhao et al. produced cube MOF-5 with a monocrystal structure of sizes in the range of 40–60 µm^43^.

Figure 1 (right) shows the FESEM image of ACS-RGO Nano sheets. The uniform and multilayer pattern for graphene Nano sheets indicates the proper synthesis and absence of salt crystals and other impurities.

**Figure 2:**
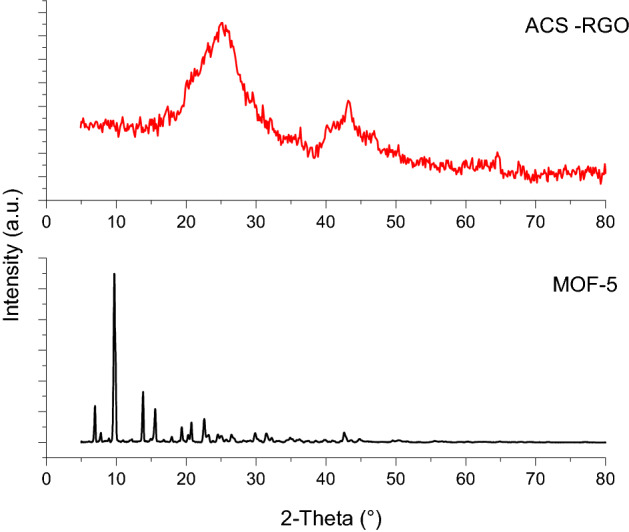
XRD patterns for as-synthesized MOF-5 and ACS-RGO.

Figure 2 shows the XRD patterns for MOF-5 and ACS-RGO. For MOF-5, all characteristic peaks at 2θ values of 6.85°, 9.69°, 13.74°, and 15.31° exist and match well to the standard pattern reported in the literature^44^. The XRD pattern for ACS-RGO shows a broad peak at 2θ values of 20 to 30° which belongs to the main index of graphite (2θ = 26°) with a space of about 3.34 Å between layers^45,46^.

**Figure 3:**
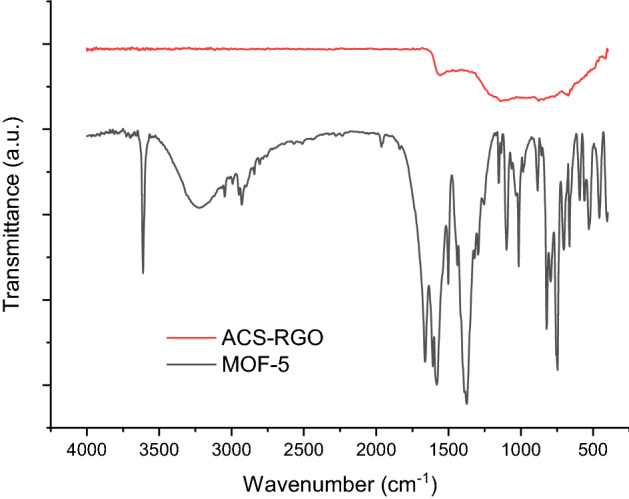
FTIR spectra for ACS-RGO, and MOF-5.

FTIR spectra for MOF-5 in Fig. 3 show chemical fingerprint peaks of this material at around 1580–1590 cm^−1^ which is indicative of asymmetric and symmetric stretching vibrations of –COO– in TPA linker. Also, the sharp peak at around 1506 cm^−1^ can be attributed to C=C stretching vibration in the linker. The graph also shows two characteristic bands at 826 and 724 cm^−1^ that are linked to C-H vibration in out-of-plane deformation vibration in MOF-5 crystals^47^.

The broad peak at 3400 1/cm can be ascribed to the presence of hydroxyl groups on the ACS-RGO surface. Two peaks at 1080 cm^−1^ and 1339 cm^−1^ belong to the vibrations of C-O and C–OH in the structure of ACS-RGO, respectively. The characteristic peak at 1367 cm^−1^ can be attributed to the stretching vibrations of C-N^48^.

Due to the adsorption of MG into the pores of MOF-5, the interaction between the Zn metal node and the organic linker moieties becomes weaker. The peaks corresponding to the symmetric and asymmetric vibrations of carboxyl groups (-COOH) present in the organic ligands of MOF-5 appear at 1374 cm^−1^ and 1598 cm^−1^ in the MOF-5 before adsorption of MG. Due to the confinement of MG in the pores of MOF-5, the peaks of carboxylate modes are shifting towards lower wavenumber . The peak at 1370 cm^−1^ shifts to 1349 cm^−1^ and the peak 1598 cm^−1^ shifts to 1569 cm^−1^ ^49^. Similarity of FT-IR pattern for ACS-RGO before adsorption MG and after, demonstrate the adsorption of MG in the pores of ACS-RGO.

Table 1summarizesthe information related to the BET specific surface area (SSA), total pore volume, and mean pore diameter(nm) for MOF-5 and ACS-RGO. SSA is an important factor related to surface phenomena such as adsorption. The synthesis condition, source of inorganic metals, the ratio of metal/linker, type of solvent, post-synthesis solvent extraction, the presence of crystallographic defects, and other physiochemical parameters are determinant factors for SSA and other characteristics for MOFs. The common SSA reported for MOF-5 synthesized by DMF were in the range of 600–1300 m^2^/g^50^, and in some cases up to 1937 m^2^/g^47^, 2500 m^2^/g^43^, 2510 m^2^/g^22^, and 2763 m^2^/g^51^. Less SSA and pore volume for ACS-RGO are related to the 2D nature of graphene and the SSA value reported here is close to the commercial graphene oxide of Sigma-Aldrich (450 m^2^/g).

**Table 1:** The physicochemical properties for MOF-5 and ACS-RGO.

Material	Mean pore diameter (nm)	Total pore volume (cm^3^/g)	SSA (m^2^/g)
MOF-5	4.2	0.271	507.4
ACS-RGO	2.79	0.273	389.0

### Kinetic study

**Figure 4:**
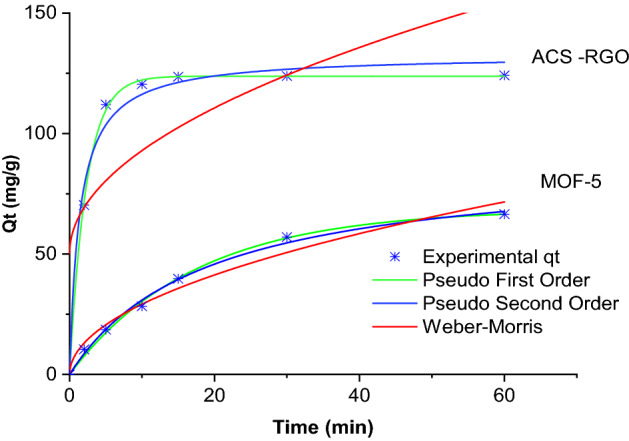
Kinetic models for MG removal by MOF-5 and ACS-RGO (MG: 50 mg/L, adsorbent: 0.4 g/L).

In the screening analysis, the efficacy of MOF-5 and ACS-RGO for dye removal was determined using adsorbent capacity and kinetic parameters. The experiments were conducted in the presence of 0.4 g/L of adsorbent in solutions containing50 mg/L MG. Dye removal was monitored for up to 60 min. The data modeled by non-linear kinetic models are described elsewhere^52^ and the results are shown in Fig. 4 and Table 2. As seen, the kinetic rate constants of MG removal by ACS-RGO was by far higher than those for MOF-5. Adsorbent capacity for MOF-5 and ACS-RGO after 60 min contact time were estimated68.6 mg/g and 123.8 mg/g, respectively. Hence, ACS-RGO was superior for MG removal compared to MOF-5. Moreover, according to statistical parameters i.e. a higher ${\mathrm{R}}_{\mathrm{Adj}}^{2}$ and R^2^, and a lower residual sum of squares (RSS) and reduced Chi-Sqr, the kinetic of MG adsorption for both adsorbents fitted well by the pseudo-first-order model. The higher adsorption for ACS-RGO could attributed to the low pH.

**Table 2:** Kinetic model parameters for MG removal by MOF-5 and ACS-RGO.

MOF-5		ACS-RGO	
Parameter	Value	Parameter	Value
**Pseudo-first order**			
q_e_ (mg/g)	68.60	q_e_ (mg/g)	123.8
k_1_ (1/min)	0.06	k_1_ (1/min)	0.43
${\mathrm{R}}_{\mathrm{Adj}}^{2}$	0.99	${\mathrm{R}}_{\mathrm{Adj}}^{2}$	0.99
R^2^	0.99	R^2^	0.99
RSS	13.99	RSS	10.85
Reduced Chi-Sqr	2.79	Reduced Chi-Sqr	2.17
**Pseudo-second order**			
q_e_ (mg/g)	88.85	q_e_ (mg/g)	132.61
k_2_ (g/mg min)	5.98	k_2_ (g/mg min)	0.005
${\mathrm{R}}_{\mathrm{Adj}}^{2}$	0.99	${\mathrm{R}}_{\mathrm{Adj}}^{2}$	0.98
R^2^	0.99	R^2^	0.98
RSS	17.21	RSS	191.34
Reduced Chi-Sqr	3.44	Reduced Chi-Sqr	38.26
**Interparticle diffusion**			
k_3_	9.26	k_3_	13.54
${\mathrm{R}}_{\mathrm{Adj}}^{2}$	0.96	${\mathrm{R}}_{\mathrm{Adj}}^{2}$	0.48
R^2^	0.97	R^2^	0.56
RSS	95.56	RSS	5668.64
Reduced Chi-Sqr	18.91	Reduced Chi-Sqr	1333.72

### Parametric study

**Figure 5:**
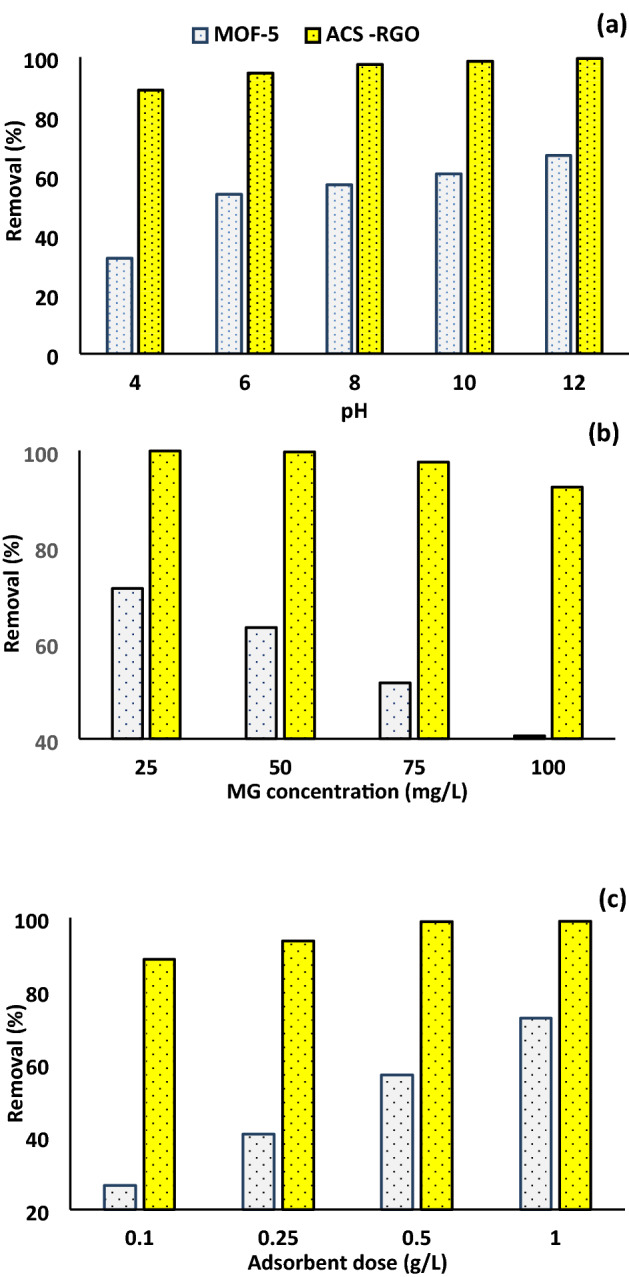
Parametric study of MG removal by ACS-RGO and MOF-5, (**a**) pH, (**b**) MG concentration, and (**c**) adsorbent dose.

Having information on the effect of operating variables is critical in optimizing the process for the highest efficiency. In adsorption systems, the pH of the solution, the concentration of pollutants, and adsorbent dose are important variables to be studied. The effect of pH in the range of 4 to12, MG concentration in the range of 25–100 mg/L, and adsorbent dose in the range of 0.1–1 g/L were studied and the results are shown in Fig. 5(a-c). As seen, in all cases, the efficacy of ACS-RGO is by far higher than MOF-5 for MG removal.

pH is an important factor governing the adsorption system through affecting the adsorbent and adsorbate charge, the level of hydroxyl and hydrogen ions, and also the charge of co-existence species in the aqueous environment. Figure 5(a) shows the effect of solution pH on dye removal by MOF-5 and ACS-RGO. The figure illustrates that the increasing pH from 4 to 12 improved MG removal by both adsorbents. However higher pH is favor for dye removal by ACS-RGO, the removal efficiency remained high (> 94%) in a wide range of pH from 6 to 12. For MOF-5, MG removal increased from ~32 to ~67% by increasing pH from 4 to 12. As a cationic dye, MG exists in aqueous environments in cationic form. The elevated dye adsorption by pH could be attributed to the surface charge of adsorbent materials and MG ionic form. pH for MOF-5 and ACS-RGO were 4.6 and 8.3, respectively. The surface charge of adsorbents turns negative at pH values above the pH, and hence a predominant electrostatic attraction force enhances the MG removal. Incremental removal by pH was observed in MG removal by chemically modified rice husk^53^, natural zeolite^54^, and reduced graphene oxide^55^. In some of these studies, removal percentage keeps constant at pH values over an optimum level.

Dye concentration is also an important factor determining the adsorption efficiency. Figure 5(b) shows adsorption removal was dye concentration-dependent for both adsorbents. The removal efficiency for MOF-5 and ACS-RGO decreased from ~ 71% to about 40% and from ~ 99% to about 92% by escalating MG concentration from 25 to 100 mg/L. The high affinity of ACS-RGO causes a premium dye uptake even at concentrated solutions. Lower adsorption rate at high concentrations is attributed to the competition between MG ions for infinite adsorption sites on the surface. Adsorption may also hinder by limitation in mass transfer rate at the higher dye concentration. It is noticeable that contrary to adsorption efficiency, the adsorbent capacity increased dramatically by dye concentration in the studied range, from ~ 44 to ~ 101 mg/g, and from ~ 62.4 to ~ 231 mg/g for MOF-5 and ACS-RGO, respectively. Similar observations were reported for dye eriochrome black-T removal onto ZIF-67-OAc^56^, caffeine removal by oxidized carbon^57^, Cr(VI) removal by fly ash^58^, antibiotics^59^ removals by MOFs, direct Blue-71 removal onto multi-walled carbon nanotubes^60^, and in advance oxidation processes such as petroleum hydrocarbons degradation by ozonation^61^.

Mass of adsorbent applied to the system is another important variable that provides the removal sites for the sorbate. Dose of MOF-5 and ACS-RGO between 0.1–1 g/L was investigated and the results are shown in Fig. 5(c). MG removal by ACS-RGO was almost complete at doses above 0.4 g/L and the removal decreased to ~ 89% by decreasing dose to 0.1 g/L. Interestingly, the dye removal occurs rather fast in a few tens of seconds after adding ACS-RGO at doses above 0.5 g/L. Dye adsorption by MOF-5 happened slowly and removal efficiencies decreased from ~ 72% to ~ 26% by decreasing mass from 1 to 0.1 g/L. Increased adsorption by dose was reported for heavy metals removal by low-cost biosorbents^62^.

### Isotherm modeling

Adsorption isotherms are mathematical models describing the system behavior when the adsorbate/adsorbent reaches the equilibrium state. These mathematical models are useful tools to estimate basic parameters for the design and operation of real adsorption units. In this study the equilibrium data were obtained experimentally by performing the experiments at pH = 10, ACS-RGO dose = 0.2 g/L, MOF-5 = 1 g/L, mixing time = 120 min, and five different initial MG concentrations varied from50–300 mg/L. The data first fitted to linear form to have an estimation of isotherm parameters. Since non-linear regression is preferable and gives a more accurate estimation of model parameters^63^, they applied to the experimental equilibrium data.

**Figure 6:**
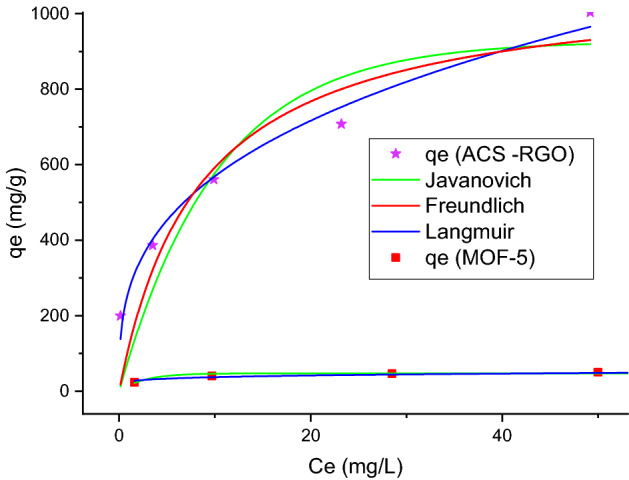
The illustration of isotherm models applied to equilibrium MG removal by ACS-RGO and MOF-5.

The non-linear form of isotherm models that are of two parametric classes are given in Table S1^52^. The illustration of isotherm models and values obtained for adsorbents are presented in Fig. 6 and Table 3, respectively.

**Table 3:** The values of isotherm parameters for MG removal by ACS-RGO and MOF-5.

Isotherm	Parameters	Value	
		ACS-RGO	MOF-5
Langmuir	b (L/mg)	0.11	0.49
	q_e_ (mg/g)	1088.27	50.69
	RSS	52,029.88	6.37
	${\mathrm{R}}_{\mathrm{Adj}}^{2}$	0.81	0.98
	R^2^	0.86	0.98
Freundlich	K_f_ (mg/g)/(mg)^1/n^	265.77	25.58
	n	3.02	6.16
	RSS	7573.29	51.64
	${\mathrm{R}}_{\mathrm{Adj}}^{2}$	0.97	0.86
	R^2^	0.98	0.89
Jovanovic	q_m_ (mg g^‒1^)	926.92	47.24
	K_j_ (L mg^‒1^)	-0.09	-0.38
	RSS	71,293.50	53.78
	${\mathrm{R}}_{\mathrm{Adj}}^{2}$	0.74	0.85
	R^2^	0.81	0.89

The maximum monolayer adsorption capacity (qmax) estimated by the Langmuir model is a useful tool to compare the economic feasibility of different adsorbents toward a specific contaminant. The qmax values for MG reported for carbonaceous materials and MOF-based adsorbents are presented in Table 4. As seen, the ACS-RGO is superior to many reported carbon-based adsorbents. Present study highlighted the significant role of surface modification of carbon-based materials to improve their adsorptive properties.

**Table 4:** Comparison of qmax for MG for carbon-based and MOF-based adsorbents.

Carbonaceous adsorbents	Qmax mg/g	Refs.	MOF adsorbents	Qmax mg/g	Refs.
Biochar supported nzvi composite (nzvi/BC)	515	^64^	ZIF-67	3000	^65^
Activated carbon with multimodal pore size distribution	144.3	^66^	UiO-66	133	^67^
Cellulose nanofibril aerogels	212.7	^68^	Fe-BTC MOF	177	^69^
Magnetic graphene oxide decorated with persimmon tannins (Fe3O4/PT/GO)	591.7	^70^	MIL-10-SO3H	596	^71^
Chitosan/polyacrylic acid/bentonite composites (CCS/PAA/bnts)	384.62–454.55	^72^	MIL-100(Fe)	485	^73^
Fe–Mg bimetallic magnetic activated carbon	4031.9	^74^	MIL-53(Al)-NH2	141	^75^
Mesoporous activated biochar (ABC)	1341	^76^	Magnetic NH2-MIL-101(Al)	274.4	^77^
Graphene oxide/aminated lignin aerogels	113.5	^78^	Fe3O4@AMCA-MIL-53(Al) nanocomposite	328.4	^79^
ACS-RGO	1088.27	This study	MOF-5	50.69	This study

The adsorption capacity of MOF-5, on the other hand, was not significant among the studied MOF-base materials. A challenging issue in the application of some MOFs is the structural stability of these materials in aqueous environment. This is a case for MOF-5 that has a metastable structure in water medium. Therefore, a sample of a MOF may have a high adsorption capacity, but during the adsorption and as a result of agitation dissolved gradually and leached the adsorbed dye into the solution.

### Regeneration study of ACS-RGO

**Figure 7:**
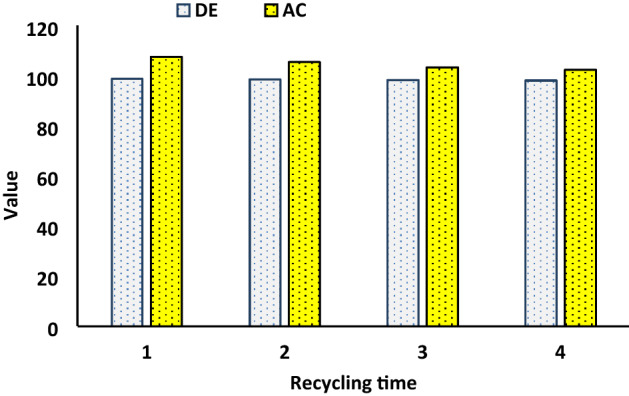
The illustration of desorption efficiency (DE) and adsorption capacity (× 10^−1^) (AC) parameters under use/reuse cycles of ACS-RGO.

Figure 7 present the result of four series of regeneration/reuse of ACS-RGO. Dilute hydrochloric acid was used as the eluting agent to desorb MG. As seen in the Fig. 7, after four cycles of regeneration and reuse, the adsorption capacity of ACS-RGOMG reduced only 6.1%. The insignificant loss in removal efficiency proved that the predominant mechanism for MG removal by ACS-RGO was being ion exchange. Moreover, the reusability of ACS-RGO indicated the promising nature of ACS-RGO to alleviate the nuisances of dyes in the environment.

## Conclusion

In this study, two adsorbent materials, i.e. MOF-5 and aminated corn Stover reduced graphite oxide (ACS-RGO) of typical adsorbents of metal–organic-frameworks (MOFs) and carbon-based classes were studied for cationic MG adsorption. MOF-5 and ACS-RGO had a specific surface area and total pore volume of 507.4 and 389.0 m^2^/g, and 0.271 cm^3^/g and 0.273 cm^3^/g, respectively. ACS-RGO was superior for MG adsorption and the kinetic rate and adsorption capacity for ACS-RGO was ~ 7.2 and ~ 21 times compared to MOF-5. For ACS-RGO, MG removal remained high (> 94%) in a wide range of ph. Dye removal onto MOF-5 increased from ~ 32% to ~ 67% by increasing pH from 4 to 12. Increasing dye from 25 mg/L to 100 mg/L decreased adsorption by MOF-5 and ACS-RGO for ~ 30% and 7%, respectively. Dye removal was rather fast and significant removal was observed in a few tens of seconds after adding ACS-RGO. Multilayer adsorption with a huge adsorption capacity of 1088.27 mg/g described MG adsorption on to ACS-RGO.

## Supplementary Information

Supplementary Information.

## Data Availability

The datasets generated and analysed during the current study available from the corresponding author on reasonable request.

## References

[1] Garg VK, Gupta R, Bala Yadav A, Kumar R (2003). Dye removal from aqueous solution by adsorption on treated sawdust. Bioresour. Technol..

[2] Salleh MAM, Mahmoud DK, Karim WAWA, Idris A (2011). Cationic and anionic dye adsorption by agricultural solid wastes: A comprehensive review. Desalination.

[3] Hameed BH, El-Khaiary M (2008). Equilibrium, kinetics and mechanism of malachite green adsorption on activated carbon prepared from bamboo by K_2_CO_3_ activation and subsequent gasification with CO_2_. J. Hazard. Mater..

[4] Culp SJ (1999). Toxicity and metabolism of malachite green and leucomalachite green during short-term feeding to Fischer 344 rats and B6C3F1 mice. Chem. Biol. Interact..

[5] Al-Ghouti MA, Sweleh AO (2019). Optimizing textile dye removal by activated carbon prepared from olive stones. Environ. Technol. Innov..

[6] Meng F (2012). Visible light photocatalytic activity of nitrogen-doped La_2_Ti_2_O_7_ nanosheets originating from band gap narrowing. Nano Res..

[7] Guo J, Jiang D, Wu Y, Zhou P, Lan Y (2011). Degradation of methyl orange by Zn(0) assisted with silica gel. J. Hazard. Mater..

[8] Xu L, Du L-S, Wang C, Xu W (2012). Nanofiltration coupled with electrolytic oxidation in treating simulated dye wastewater. J. Membr. Sci..

[9] Palanisamy S (2020). Application of electrochemical treatment for the removal of triazine dye using aluminium electrodes. J. Water Supply Res. Technol. AQUA.

[10] Bhatia D, Sharma NR, Singh J, Kanwar RS (2017). Biological methods for textile dye removal from wastewater: A review. Crit. Rev. Environ. Sci. Technol..

[11] Yagub MT, Sen TK, Afroze S, Ang HM (2014). Dye and its removal from aqueous solution by adsorption: A review. Adv. Coll. Interface. Sci..

[12] Kadhom M, Albayati N, Alalwan H, Al-Furaiji M (2020). Removal of dyes by agricultural waste. Sustain. Chem. Pharm..

[13] Acar ET, Ortaboy S, Atun G (2015). Adsorptive removal of thiazine dyes from aqueous solutions by oil shale and its oil processing residues: Characterization, equilibrium, kinetics and modeling studies. Chem. Eng. J..

[14] Pathania D, Sharma S, Singh P (2017). Removal of methylene blue by adsorption onto activated carbon developed from *Ficus carica* bast. Arab. J. Chem..

[15] Wang S, Zhu Z (2006). Characterisation and environmental application of an Australian natural zeolite for basic dye removal from aqueous solution. J. Hazard. Mater..

[16] Valizadeh B, Nguyen TN, Stylianou KC (2018). Shape engineering of metal–organic frameworks. Polyhedron.

[17] Oladoye PO, Adegboyega SA, Giwa A-RA (2021). Remediation potentials of composite metal-organic frameworks (MOFs) for dyes as water contaminants: A comprehensive review of recent literatures. Environ. Nanotechnol. Monit. Manag..

[18] Chen G (2019). Investigation of metal-organic framework-5 (MOF-5) as an antitumor drug oridonin sustained release carrier. Molecules.

[19] Burgaz E, Erciyes A, Andac M, Andac O (2019). Synthesis and characterization of nano-sized metal organic framework-5 (MOF-5) by using consecutive combination of ultrasound and microwave irradiation methods. Inorg. Chim. Acta.

[20] Xu S (2020). Metal–organic framework-5 as a novel phosphorescent probe for the highly selective and sensitive detection of Pb (II) in mussels. Sens. Actuators B Chem..

[21] Kassaoui ME, Lakhal M, Benyoussef A, El Kenz A, Loulidi M (2021). Effect of zinc substitution by magnesium and cadmium on hydrogen storage properties of connector-metal-organic framework-5. J. Alloys Compd..

[22] Mirsoleimani-azizi SM, Setoodeh P, Zeinali S, Rahimpour MR (2018). Tetracycline antibiotic removal from aqueous solutions by MOF-5: Adsorption isotherm, kinetic and thermodynamic studies. J. Environ. Chem. Eng..

[23] Mehmandoust MR, Motakef-Kazemi N, Ashouri F (2019). Nitrate adsorption from aqueous solution by metal–organic framework MOF-5. Iran. J. Sci. Technol. Trans. A Sci..

[24] Mohammed MTE, Djamel N, Mohamed T, Amokrane S (2021). Study of the adsorption of an organic pollutant onto a microporous metal organic framework. Water Sci. Technol..

[25] Wu Y (2018). Synthesis of rod-like metal-organic framework (MOF-5) nanomaterial for efficient removal of U (VI): Batch experiments and spectroscopy study. Sci. Bull..

[26] Prekodravac J, Kepic D, Colmenares JC, Giannakoudakis DA, Jovanovic SP (2021). A comprehensive review on selected graphene synthesis methods: From electrochemical exfoliation through rapid thermal annealing towards biomass pyrolysis. J. Mater. Chem. C.

[27] Sadeghi S (2021). Modified wheat straw-derived graphene for the removal of Eriochrome Black T: Characterization, isotherm, and kinetic studies. Environ. Sci. Pollut. Res..

[28] Javid A (2020). Modeling of chromium (VI) removal from aqueous solution using modified green-Graphene: RSM-CCD approach, optimization, isotherm, and kinetic studies. J. Environ. Health Sci. Eng..

[29] Liu X (2019). Graphene oxide-based materials for efficient removal of heavy metal ions from aqueous solution: A review. Environ. Pollut..

[30] Ahmad H, Fan M, Hui D (2018). Graphene oxide incorporated functional materials: A review. Compos. B Eng..

[31] Wang J, Chen B (2015). Adsorption and coadsorption of organic pollutants and a heavy metal by graphene oxide and reduced graphene materials. Chem. Eng. J..

[32] Wanjeri VWO, Sheppard CJ, Prinsloo ARE, Ngila JC, Ndungu PG (2018). Isotherm and kinetic investigations on the adsorption of organophosphorus pesticides on graphene oxide based silica coated magnetic nanoparticles functionalized with 2-phenylethylamine. J. Environ. Chem. Eng..

[33] Yao N (2020). Insight into adsorption of combined antibiotic-heavy metal contaminants on graphene oxide in water. Sep. Purif. Technol..

[34] Gao Y (2012). Adsorption and removal of tetracycline antibiotics from aqueous solution by graphene oxide. J. Colloid Interface Sci..

[35] Fang F (2014). Removal of cobalt ions from aqueous solution by an amination graphene oxide nanocomposite. J. Hazard. Mater..

[36] Awad FS, AbouZied KM, Abou El-Maaty WM, El-Wakil AM, El-Shall MS (2020). Effective removal of mercury (II) from aqueous solutions by chemically modified graphene oxide nanosheets. Arab. J. Chem..

[37] Viana MM (2020). Microwave-assisted synthesis of polyacrylamide-aminated graphene oxide hybrid hydrogel with improved adsorption properties. J. Environ. Chem. Eng..

[38] Haghighat GA (2020). Aminated graphitic carbon derived from corn stover biomass as adsorbent against antibiotic tetracycline: Optimizing the physicochemical parameters. J. Mol. Liq..

[39] Zhao X-H (2018). Multifunctional sensor based on porous carbon derived from metal–organic frameworks for real time health monitoring. ACS Appl. Mater. Interfaces.

[40] Saranya N, Ajmani A, Sivasubramanian V, Selvaraju N (2018). Hexavalent Chromium removal from simulated and real effluents using *Artocarpus heterophyllus* peel biosorbent—Batch and continuous studies. J. Mol. Liq..

[41] Son W-J, Kim J, Kim J, Ahn W-S (2008). Sonochemical synthesis of MOF-5. Chem. Commun..

[42] Choi J-S, Son W-J, Kim J, Ahn W-S (2008). Metal–organic framework MOF-5 prepared by microwave heating: Factors to be considered. Microporous Mesoporous Mater..

[43] Zhao Z, Li Z, Lin Y (2009). Adsorption and diffusion of carbon dioxide on metal–organic framework (MOF-5). Ind. Eng. Chem. Res..

[44] Li H, Eddaoudi M, O'Keeffe M, Yaghi OM (1999). Design and synthesis of an exceptionally stable and highly porous metal-organic framework. Nature.

[45] Giannakoudakis DA (2020). Ultrasound-activated TiO_2_/GO-based bifunctional photoreactive adsorbents for detoxification of chemical warfare agent surrogate vapors. Chem. Eng. J..

[46] Bonyadi Z (2021). Biomass-derived porous aminated graphitic nanosheets for removal of the pharmaceutical metronidazole: Optimization of physicochemical features and exploration of process mechanisms. Colloids Surf. A.

[47] Biserčić MS (2019). The quest for optimal water quantity in the synthesis of metal–organic framework MOF-5. Microporous Mesoporous Mater..

[48] Ghadiri SK (2017). Adsorption of nitrate onto anionic bio-graphene nanosheet from aqueous solutions: Isotherm and kinetic study. J. Mol. Liq..

[49] Dhumal NR, Singh MP, Anderson JA, Kiefer J, Kim HJ (2016). Molecular interactions of a Cu-based metal-organic framework with a confined imidazolium-based ionic liquid: A combined density functional theory and experimental vibrational spectroscopy study. J. Phys. Chem. C.

[50] Tsao C-S (2007). Characterization of pore structure in metal–organic framework by small-angle X-ray scattering. J. Am. Chem. Soc..

[51] Ming Y (2014). Thermophysical properties of MOF-5 powders. Microporous Mesoporous Mater..

[52] Shams M (2021). Parameter optimization of tetracycline removal by vanadium oxide nano cuboids. Colloids Surf. A.

[53] Chowdhury S, Mishra R, Saha P, Kushwaha P (2011). Adsorption thermodynamics, kinetics and isosteric heat of adsorption of malachite green onto chemically modified rice husk. Desalination.

[54] Wang S, Ariyanto E (2007). Competitive adsorption of malachite green and Pb ions on natural zeolite. J. Colloid Interface Sci..

[55] Gupta K, Khatri OP (2017). Reduced graphene oxide as an effective adsorbent for removal of malachite green dye: Plausible adsorption pathways. J. Colloid Interface Sci..

[56] Haghighat GA (2020). Zeolitic imidazolate frameworks (ZIFs) of various morphologies against eriochrome black-T (EBT): Optimizing the key physicochemical features by process modeling. Colloids Surf. A.

[57] Anastopoulos I, Pashalidis I (2019). Τhe application of oxidized carbon derived from *Luffa cylindrica* for caffeine removal. Equilibrium, thermodynamic, kinetic and mechanistic analysis. J. Mol. Liq..

[58] Jahangiri K (2018). Enhancement adsorption of hexavalent chromium onto modified fly ash from aqueous solution; optimization; isotherm, kinetic and thermodynamic study. J. Dispersion Sci. Technol..

[59] Nezhad NT (2021). Vanadium dioxide nanoparticles as a promising sorbent for controlled removal of waterborne fluoroquinolone ciprofloxacin. Mater. Chem. Phys..

[60] Fard RF (2018). Efficiency of multi walled carbon nanotubes for removing Direct Blue 71 from aqueous solutions. Eurasian J. Anal. Chem..

[61] Mehrizi EA, Kermani M, Farzadkia M, Esarfili A, Ghorbanian M (2019). Study of improvement of bioremediation performance for the degradation of petroleum hydrocarbons in oily sludge by a chemical pretreatment strategy. J. Mater. Cycles Waste Manag..

[62] Abatal M (2021). Comparison of heavy metals removal from aqueous solution by *Moringa oleifera* leaves and seeds. Coatings.

[63] Liakos EV (2021). Activated porous carbon derived from tea and plane tree leaves biomass for the removal of pharmaceutical compounds from wastewaters. Antibiotics.

[64] Eltaweil A, Mohamed HA, Abd El-Monaem EM, El-Subruiti G (2020). Mesoporous magnetic biochar composite for enhanced adsorption of malachite green dye: Characterization, adsorption kinetics, thermodynamics and isotherms. Adv. Powder Technol..

[65] Lin K-YA, Chang H-A (2015). Ultra-high adsorption capacity of zeolitic imidazole framework-67 (ZIF-67) for removal of malachite green from water. Chemosphere.

[66] Bagheri R, Ghaedi M, Asfaram A, Alipanahpour Dil E, Javadian H (2019). RSM-CCD design of malachite green adsorption onto activated carbon with multimodal pore size distribution prepared from *Amygdalus scoparia*: Kinetic and isotherm studies. Polyhedron.

[67] Ahmadijokani F (2020). Superior chemical stability of UiO-66 metal-organic frameworks (MOFs) for selective dye adsorption. Chem. Eng. J..

[68] Jiang F, Dinh DM, Hsieh Y-L (2017). Adsorption and desorption of cationic malachite green dye on cellulose nanofibril aerogels. Carbohydr. Polym..

[69] Delpiano GR, Tocco D, Medda L, Magner E, Salis A (2021). Adsorption of malachite green and alizarin red s dyes using fe-btc metal organic framework as adsorbent. Int. J. Mol. Sci..

[70] Gao M (2019). Novel magnetic graphene oxide decorated with persimmon tannins for efficient adsorption of malachite green from aqueous solutions. Colloids Surf. A.

[71] Luo X-P, Fu S-Y, Du Y-M, Guo J-Z, Li B (2017). Adsorption of methylene blue and malachite green from aqueous solution by sulfonic acid group modified MIL-101. Microporous Mesoporous Mater..

[72] Yildirim A, Bulut Y (2020). Adsorption behaviors of malachite green by using crosslinked chitosan/polyacrylic acid/bentonite composites with different ratios. Environ. Technol. Innov..

[73] Huo S-H, Yan X-P (2012). Metal–organic framework MIL-100 (Fe) for the adsorption of malachite green from aqueous solution. J. Mater. Chem..

[74] Guo F (2020). Synthesis of MgO/Fe_3_O_4_ nanoparticles embedded activated carbon from biomass for high-efficient adsorption of malachite green. Mater. Chem. Phys..

[75] Li C, Xiong Z, Zhang J, Wu C (2015). The strengthening role of the amino group in metal–organic framework MIL-53 (Al) for methylene blue and malachite green dye adsorption. J. Chem. Eng. Data.

[76] Choudhary M, Kumar R, Neogi S (2020). Activated biochar derived from *Opuntia ficus*-indica for the efficient adsorption of malachite green dye, Cu+2 and Ni+2 from water. J. Hazard. Mater..

[77] Liu H, Chen L, Ding J (2016). Adsorption behavior of magnetic amino-functionalized metal–organic framework for cationic and anionic dyes from aqueous solution. RSC Adv..

[78] Chen H (2020). Novel graphene oxide/aminated lignin aerogels for enhanced adsorption of malachite green in wastewater. Colloids Surf. A.

[79] Alqadami AA, Naushad M, Alothman ZA, Ahamad T (2018). Adsorptive performance of MOF nanocomposite for methylene blue and malachite green dyes: Kinetics, isotherm and mechanism. J. Environ. Manag..

