# Supplementary material for: Comparative removal of hazardous cationic dyes by MOF-5 and modified graphene oxide
Source: Sci Rep. 2022 Sep 12;12:15314. doi: 10.1038/s41598-022-19550-5 (PMC9468029; doi:10.1038/s41598-022-19550-5)
Supplement: Supplementary file 1 — Supplementary Information. [file 41598_2022_19550_MOESM1_ESM.docx]

***Supplementary data***

**Comparative removal of hazardous cationic dyes by MOF-5 and modified graphene oxide**

**
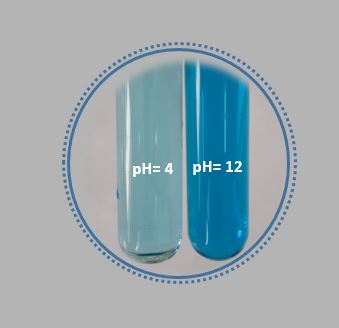
**

Fig S1. Dye intensity of a 50 mg/L MG at pH 4 and pH 12.

**Table S1**. Non-linear isotherm models fitted to equilibrium data for for MG removal by ACS -RGO and MOF-5

| Isotherm | Equation | Parameters |
| --- | --- | --- |
| Two-parameter models | | |
| Langmuir | $q_{e}= \frac{Q_{0}K_{L}C_{e}}{1+ K_{L}C_{e}}$ | C_e_= adsorbate equilibrium concentration (mg/L)  q_e_ = adsorption capacity at equilibrium (mg/g)  Q_0_= monolayer coverage capacity (mg/g)  K_L_= Langmuir isotherm constant (L/mg). |
| Freundlich | $q_{e}= K_{f}C_{e}^{1/n}$ | K_f_ = Freundlich isotherm constant(mg^1-(1/n)^ L^1/n^ g^-1^)  n = adsorption intensity |
| Jovanovic | $q_{e}= q_{m}\left( 1-e^{K_{j}C_{e}} \right)$ | K_j_ = Jovanovic isotherm constant(L/mg)  q_m_= maximum adsorption capacity(mg/g) |
